# Synergistic combination of next-generation polymyxin MRX-8 and meropenem against carbapenem-resistant *A. baumannii*

**DOI:** 10.1128/aac.01912-24

**Published:** 2025-05-27

**Authors:** Xiaofen Liu, Xingyi Qu, Ruohao Zhang, Yuxin Zhang, Xingchen Bian, Yu-Wei Lin, Jing Zhang

**Affiliations:** 1 Institute of Antibiotics, Huashan Hospital Affiliated to Fudan University, Key Laboratory of Clinical Pharmacology of the National Health Commission, National Clinical Research Center for Aging and Diseases, Shanghai, USA; 2 Clinical Pharmacology Center, Huashan Hospital Affiliated to Fudan University, Shanghai, USA; 3 Infection Program and Department of Microbiology, Monash Biomedicine Discovery Institute, Monash University 161661, Clayton, Victoria, Australia; Providence Portland Medical Center, Portland, Oregon, USA

**Keywords:** MRX-8, meropenem, carbapenem-resistant *A. baumannii*, *in vitro* pharmacokinetics/pharmacodynamics, hollow-fiber infection model

## Abstract

MRX-8 is a new next-generation polymyxin with potent antibacterial activity against carbapenem-resistant *Acinetobacter baumannii* (CRAB). This study evaluated the MRX-8 and meropenem combination and their dosing regimens against CRAB in clinics. Seven CRAB strains isolated from Huashan Hospital were tested. Two strains of AB21-3881 and AB21-3759 were selected for static time-kill and the Hollow Fiber Infection Model (HFIM). Adaptive resistance to MRX-8 was assessed via population analysis profiling (PAP) at 2 mg/L of MRX-8. Multilocus sequence typing identified all seven strains as ST2. Minimum inhibitory concentration values for MRX-8 ranged from 0.5 to 1 mg/L. Synergy was observed in six out of seven (85.7%) strains. The combination of 1 mg/L MRX-8 with 1 mg/L meropenem completely inhibited bacterial growth within 24 h for both selected strains. In HFIM, the combination of MRX-8 (0.75 mg/kg q8h continuous infusion) and meropenem (1 g q6h continuous infusion) achieved synergistic killing over 72 h for AB21-3759, while single treatment of MRX-8 (0.75 mg/kg q8h continuous infusion) achieved bactericidal effect (lower than the detect limitation) over 72 h. PAP analysis demonstrated that the combinational therapy could delay the emergence of adaptive resistance sub-populations by 12–24 h. The combination of MRX-8 and meropenem demonstrated synergistic bactericidal activity by checkerboard and static time-kill curves. Additionally, in HFIM, MRX-8 at 1 mg/kg q12h combined with meropenem at 2 g q12h, as well as MRX-8 at 0.75 mg/kg q8h continuous infusion combined with meropenem at 1 g q6h continuous infusion, achieved bacteriostatic killing at 72 h compared to the initial inoculum.

## INTRODUCTION

Infections caused by carbapenem-resistant organisms (CRO) pose a significant challenge to global public health, with the mortality rates reaching as high as 30–40% (1). Of particular concern is the emergence of carbapenem-resistant *Acinetobacter baumannii* (CRAB), which has shown an alarming rise in prevalence year by year. Surveillance data from China indicate that the resistance rate of CRAB reached 80% in 2023 (https://www.chinets.com). CRAB primarily confers resistance by producing oxacillinase (OXA), such as OXA-23, OXA-51, and OXA-58 (2). Unfortunately, there are limited therapeutic options for treating CRAB infections. Polymyxins are important options for CRAB, which has a high susceptible rate of 98.2% (https://www.chinets.com/Data/AntibioticDrugFast). Novel β-lactamase inhibitors (avibactam, relebactam, or vaborbactam) are ineffective against OXA (3). Studies on durlobactam, which has shown efficacy against class D OXA, indicate that the susceptible rate of CRAB carrying *bla*_OXA-23_ to sulbactam-durlobactam is as high as 97.7% (4). Polymyxins and sulbactam-durlobactam are last-line treatments for CRAB infections.

Currently available polymyxins, such as polymyxin B sulfate and colistimethate, are dose-limited by nephrotoxicity (5, 6). MRX-8, a next-generation polymyxin, modifies the fatty acyl tail of polymyxin B1 via an ester bond, offering a promising alternative. Previous studies demonstrated that the minimal inhibitory concentration (MIC_50_/MIC_90_) of MRX-8 against clinical strains of CRO isolated in China and the United States (2017 to 2020) was equivalent to or lower than that of polymyxin B (7, 8). In mouse thigh and lung infection models, MRX-8 exhibited a lower pharmacokinetic/pharmacodynamic (PK/PD) target for *A. baumannii* and superior bactericidal activity than polymyxin B (9). Preclinical studies revealed reduced nephrotoxicity of MRX-8 in rats compared to polymyxin B (10). Phase I clinical trials conducted in the United States and China demonstrated that MRX-8 is completed. All the subjects completed the study according to the protocol, and no subject withdrew from or terminated the study due to adverse events at daily doses up to 2.5 mg/kg. The most common adverse events were mild hypoesthesia and decreased glomerular filtration rate, all of which were CTCAE grade 1 and could recover without intervention. The results indicated that the multiple doses of 2.5 mg/kg/day are safe and tolerable in healthy subjects.

Carbapenems, especially meropenem, when combined with polymyxins, have demonstrated synergistic effects against *A. baumannii in vitro* (11). The Hollow Fiber Infection Model (HFIM) is widely used to evaluate bactericidal effects under the time-concentration profiles of different dosage regimens of antibiotics in humans. HFIM is considered the “golden standard” for *in vitro* dynamic PK/PD studies of antibacterial drugs (12, 13). Compared to the traditional *in vitro* PK/PD model (13), HFIM offers a higher surface-area-to-volume ratio, enabling enhanced interaction between bacteria and antibacterial drugs. Therefore, using HFIM to investigate the bactericidal effects when simulating various dosing regimens of MRX-8 and meropenem, both alone and in combination, would provide valuable insights into the optimal dosing strategies for combination therapy with MRX-8.

This study aims to evaluate the bactericidal effects of MRX-8 and meropenem combination against CRAB and to optimize dosing regimens for this combination therapy.

## MATERIALS AND METHODS

### Reagents and bacterial strains

MRX-8, in which the free-base content is 84.11% (batch number CKo123134-01-04-01-11-01-RS), was provided by Shanghai MicuRx Pharmaceutical Co., Ltd. Meropenem (purity 86.8%, batch number 130506-202004) was obtained from National Institutes for Food and Drug Control (Shanghai, China). The cation-adjusted Muller-Hinton broth (CAMHB) medium and Luria-Bertani agar (LBA) medium were purchased from Becton, Dickinson and Company (NJ, US).

Seven strains of clinically isolated CRAB from Huashan Hospital Affiliated to Fudan University from 2019 to 2021 were selected. The multilocus sequence typing of these seven CRAB strains was ST2. All strains harbored *bla*_OXA-23_, which confers the main resistance mechanism to meropenem. Besides *bla*_OXA-23_, genome sequencing showed both strains of AB21-3759 and AB21-3881 harbored outer membrane porin (Opr), resistance-nodulation-cell division antibiotic efflux pump (adeNIJKSRABCHFLM, MexJK), ATP-binding cassette antibiotic efflux pump (macAB), and major facilitator superfamily antibiotic efflux pump. These porins and efflux pumps could mediate broad-spectrum antibiotic resistance by actively extruding antibiotics. Moreover, both strains carried resistant genes of gyrA and parCE, which conferred resistance to fluoroquinolones.

### MIC and checkerboard assay

The MIC was determined by the microdilution broth according to the 2023 edition of the Clinical and Laboratory Standards Institute (CLSI) guidelines (14). The checkerboard assay was conducted following the previously published protocol (15). Briefly, aliquots of 25 µL of different concentrations of MRX-8 and meropenem were added into 96-well plates; and 50 µL of 2× CAMHB medium containing CRAB strains at approximately 10^6^ CFU/mL was added to the plates and mixed tenderly. The results were read after incubation at 35 ± 2°C for 20–24 h.

The fractional inhibitory concentration index (FICI) was used to evaluate synergistic effects of combinations. FICI = FICI_A_ + FICI_B_, FICI_A_ = MIC value of drug A in combination / MIC value of drug A alone, FICI_B_ = MIC value of drug B in combination / MIC value of drug B alone. FICI ≤ 0.5 indicated a synergistic effect; 0.5 < FICI ≤ 1 indicated an additive effect, and >1 indicated no difference or antagonism (15).

### Static time-kill curve

Two strains of AB21-3759 and AB21-3881 were selected to conduct static time-kill curves according to previously published methods (15). Briefly, bacteria were inoculated to the CAMHB medium to a final bacterial density of approximately 10^6^ CFU/mL. After incubating the bacteria in a 37 ± 2°C shaker for 1 h, different treatment groups of drugs were added. Samples were collected at 0.5, 1, 2, 4, 6, 8, 12, and 24 h for bacterial colony counting. The treatment groups of control, single drug of MRX-8 (0.125, 0.25, 0.5, and 1 mg/L), meropenem (1 and 64 mg/L), and combination of MRX-8 and meropenem (MRX-8 concentrations at 0.125, 0.25, 0.5, and 1 mg/L combined with 1 mg/L MRX-8, respectively) were conducted in triplicates.

Bactericidal effect of the drugs was defined as a ≥3 log10 reduction in the colony counts relative to the initial inoculums (15). Compared with the most effective single drug, the colony counts of the combination group at 24 h decreased by ≥2 log10 CFU/mL were considered as synergistic effect; the colony counts of the combination group at 24 h decreased by <2 log10 CFU/mL were considered as no synergistic effect (15).

### Hollow Fiber Infection Model (HFIM)

The same two strains of AB21-3759 and AB21-3881 were further employed for HFIM. For HFIM, 200 mL of CAMHB medium was prefilled into the central compartmental chamber, and the bacteria were inoculated at a density of 10^6^ CFU/mL into the hollow fiber cartridge. The drug was added into the central compartmental chamber after 1 h of bacteria pre-incubation at the cartridge. The treatment groups included control, single drug treatment of MRX-8, meropenem, and combination of MRX-8/meropenem, and multiple dosings were treated and conducted for 72 h. For dosing regimens, intravenous infusion of MRX-8 at 1 mg/kg q12h (MRX-8_1), 1.5 mg/kg q12h (MRX-8_2), and 1.5 mg/L (MRX-8_3, corresponding to 0.75 mg/kg q8h continuous infusion) were simulated according to the pharmacokinetics in healthy subjects (unpublished data). The meropenem dosing regimen was simulated at 2 g, q12h (MEM) according to the pharmacokinetics of meropenem in Chinese healthy subjects (16). Combination regimens were intravenous infusion of MRX-8 at 1 mg/kg q12h and meropenem at 2 g q12h (COM_1) and MRX-8 at 1.5 mg/L and meropenem at 8.77 mg/L (COM_2, corresponding to the dosing regimens of MRX-8 at 0.75 mg/kg q8h continuous infusion and meropenem at 1 g q6h continuous infusion). Except for the MRX-8_3 and COM_2 regimens, the other regimens were all set as 3 h infusion for each dosing. All the drug concentrations at different dosing regimens were simulated by Phoenix WinNonlin 8.3 (Pharsight Corporation, MO, US).

Samples were collected from the hollow fiber cartridge for bacterial colony counts and from the central compartmental chamber for drug concentration validation at 0, 1.5, 3, 5, 7, 9, 12, 15, 24, 27, 36, 39, 48, 49.5, 51, 53, 55, 57, 60, and 72 h. The bactericidal effect was evaluated by the area under the bacterial kill curve (AUBC) every 24 h and from 0 to 72 h and the log change in colony counts at 72 h for different dosage regimens. The concentrations of MRX-8 and meropenem in CAMHB were determined by validated liquid chromatography with tandem mass spectrometry (LC–MS/MS) methods. The relative error (%) was calculated for the concentration measured during HFIM compared with the simulated target concentrations.

### Population analysis profile (PAP) in HFIM

In the 2023 edition of the European Committee on Antimicrobial Susceptibility Testing, the susceptibility breakpoint for colistin is defined as follows: MIC ≤ 2 mg/L indicates susceptibility, while MIC > 2 mg/L indicates resistance (14). Hence, the LBA plate containing 2 mg/L of MRX-8 was employed to screen the adaptive resistant sub-population every 12 h.

## RESULTS

### MIC and checkerboard assay

The MIC and FICI results of MRX-8 and meropenem against seven strains of CRAB are shown in Table 1. The MICs of MRX-8 were in a range of 0.5 to 1 mg/L, and the MIC of meropenem was in a range of 16 to 64 mg/L. The FICI range was 0.14–0.52 for MRX-8 and meropenem combination. According to the FICI, the combination showed synergistic effects against 6/7 (85.7%) strains and additive effect against one strain. Two strains (AB21-3759 and AB21-3881) that were highly resistant to meropenem (MIC = 64 mg/L) and showed synergistic and additive effects, respectively, for the combination were selected for the following static time-kill curve and HFIM.

**TABLE 1:** MIC and FICI by checkerboard assay

Strains	MRX-8 MIC^*a*^ (mg/L)	Meropenem MIC (mg/L)	FICI
AB1920	1	64	0.31
AB19J21063	1	16	0.25
AB19J34064	0.5	64	0.28
AB200723	0.5	64	0.50
AB201613	1	64	0.14
AB21-3759	0.5	64	0.27
AB21-3881	0.5	64	0.52

^*a*^ MIC: minimal inhibitory concentration.

### Static time-kill curve

**Fig 1:**
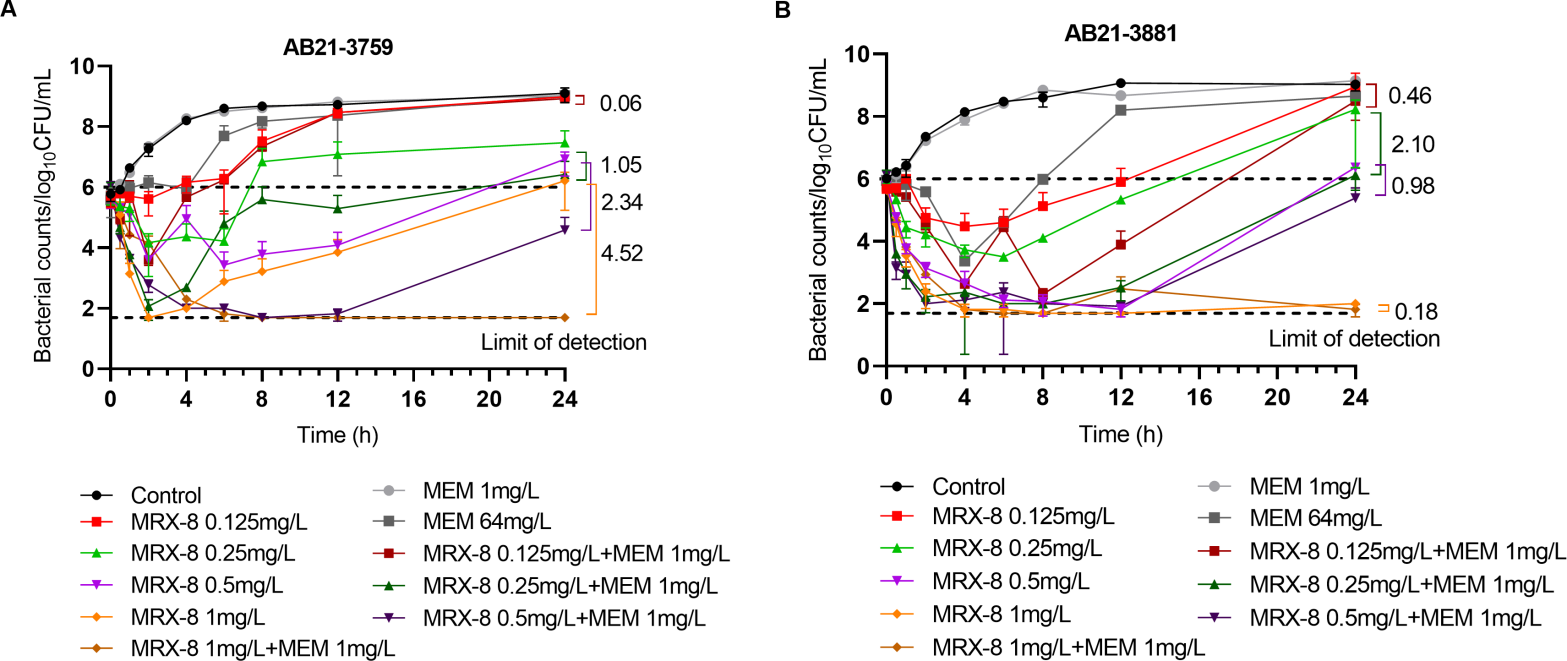
Time-kill curves of AB21-3759 (**A**) and AB21-3881 (**B**). MEM represents meropenem.

The static time-kill curves of AB21-3759 and AB21-3881 are shown in Fig. 1. For the single MRX-8 treatment, the growth of AB21-3759 was inhibited at 0 to 6 h from 0.125 to 0.25 mg/L MRX-8 treatment, and the bacterial regrowth occurred at 6 to 24 h. Among the single MRX-8 treatment groups, at MIC (MIC_MRX-8_ is 0.5 mg/L) achieved 1.0 ± 0.5 log10 CFU/mL increase compared to at initial inoculum (0 h). The 2× MIC concentration of MRX-8 treatment maintained −0.8 ± 2.5 log10 CFU/mL reduction at 24 h compared to the initial inoculum. The meropenem alone at 1 mg/L had no bactericidal effect. When meropenem at 64 mg/L had an inhibitory effect at the first 4 h on bacterial growth, and the bacteria grew to the same level as control at 24 h. The 1/2 or 1× MIC concentration of MRX-8 combined with 1 mg/L of meropenem had an additive effect. The combination of 2× MIC of MRX-8 with 1 mg/L of meropenem had a synergistic effect compared to MRX-8 alone. The combination of 2× MIC of MRX-8 with 1 mg/L of meropenem showed ~4 log10 reduction in the colony counts relative to the initial inoculum, indicating bactericidal effect and could kill the bacteria below the detection limit (1.70 log10 CFU/mL) until 24 h.

The antibacterial effect of MRX-8 alone treatment also showed a similar trend of decreasing first at 0 to 6 h, and then rebounding at 6 to 24 h for AB21-3881. Both strains had the same MICs against MRX-8; however, MRX-8 showed better bactericidal effect against AB21-3881 than AB21-3759. The treatments of MRX-8 at 0.125 to 0.25 mg/L achieved bacterial growth of −0.06 ± 3.0 to 1.8 ± 0.9 log10 CFU/mL increase at 24 h compared to initial inoculum. The treatment of 1× MIC of MRX-8 could kill the bacteria to the lower detection limit within 12 h and could achieve the bacteriostatic state at 24 h (0.3 ± 0.06 log10 CFU/mL). The treatment of 2× MIC of MRX-8 alone could kill the bacteria within 4 h and maintain the bactericidal effect for 24 h (−4.0 ± 0 log10 CFU/mL). The meropenem alone at 1 mg/L had no bactericidal effect, but at 64 mg/L, had antibacterial effect at 0 to 4 h; and the bacteria regrew to the same level as control at 24 h. The combination of 1/4 to 1× MIC of MRX-8 with 1 mg/L of meropenem could further decrease bacterial colonies compared to MRX-8 alone treatment. However, the combination achieved an additive effect against AB21-3881.

### Different dosing regimens in HFIM

**Fig 2:**
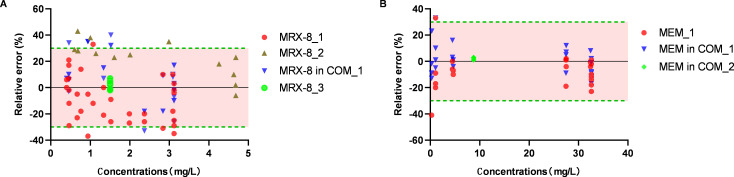
Relative errors of LC–MS/MS-measured concentrations vs. targeted concentrations for different dosing regimens of MRX-8 alone and in combination (**A**) and meropenem alone and in combination (**B**). Different symbols indicate concentrations measured in different dosing regimens.

The drug concentrations in HFIM under different dosing regimens of MRX-8 alone, meropenem alone, and the combination of MRX-8 and meropenem were determined and compared to the targeted concentrations. As shown in Fig. 2, for MRX-8 and meropenem dosing regimen simulation, 87.3 and 96.4% of the measured concentrations in HFIM fell within the 30% range of target concentrations. The PK/PD indices of AUC/MIC for MRX-8 and %T_>MIC_ for meropenem were also calculated with the measured concentrations and compared with the target indices, which were in a ±10% range. This demonstrated the drug concentrations in HFIM could well describe the pharmacokinetics of the dosing regimens.

**Fig 3:**
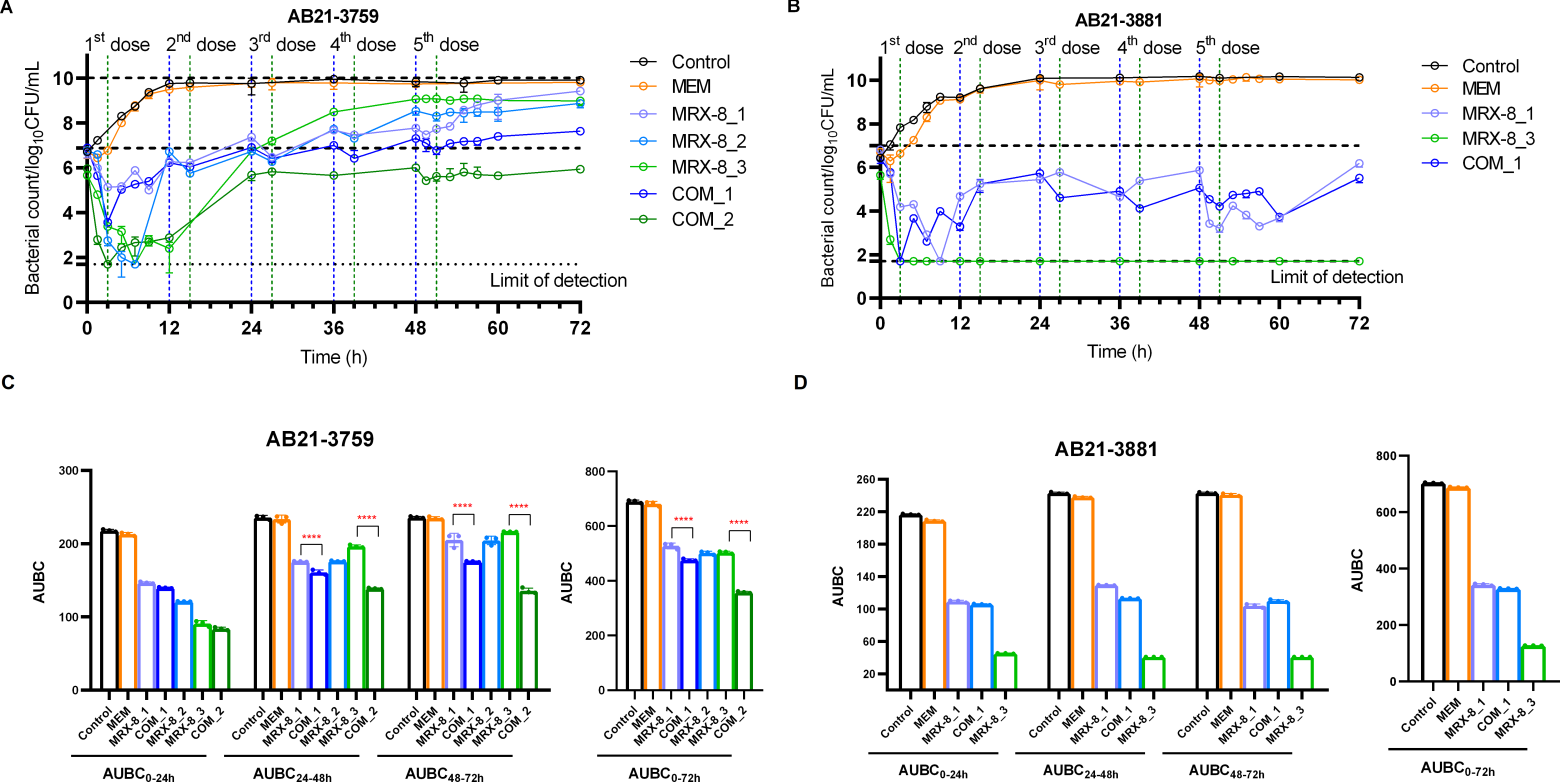
Bacterial counts and the area under the bactericidal curve (AUBC) for the two bacterial strains. AB21-3759 (**A**) and AB21-3881 (**B**) are bactericidal curves at different time points under multiple dosing regimens of MRX-8, meropenem, and the combination in HFIM. The blue dashed line represents the start of drug administration, and the green dashed line represents the end of drug administration. The AUBC for AB21-3759 (**C**) and AB21-3881 (**D**). MEM_1: meropenem 2 g q12h; MRX-8_1: MRX-8 1 mg/kg q12h; COM_1: MRX-8 1 mg/kg q12h combined with meropenem 2 g q12h; MRX-8_2: MRX-8 1.5 mg/kg q12h; MRX-8_3: MRX-8 0.75 mg/kg q8h continuous infusion; and COM_2: MRX-8 0.75 mg/kg q8h continuous infusion combined with meropenem 1 g q6h continuous infusion. ****: *P* < 0.000.

The bactericidal effects of AB21-3759 and AB21-3881 under different clinical dosing regimens of MRX-8, meropenem, and the combination investigated in HFIM were shown in Fig. 3A and B. For AB21-3759, the MRX-8 multiple dosing regimens at 1 (MRX-8_1) and 1.5 mg/kg q12h (MRX-8_2) could inhibit bacterial growth within 24 h; however, the bacteria grew to more than 8 log10 CFU/mL at 72 h. The meropenem dosing regimen at 2 g q12h could not exhibit antibacterial activity. When the combination regimen of MRX-8 at 1 mg/kg q12h and meropenem at 2 g q12h (COM_1) was given, AB21-3759 did not show a significant reduction in the number of colonies in the first 24 h compared with the MRX-8 regimen. The colonies showed 1.79 log10 CFU/mL decrease than that of MRX-8_1 regimen but increased by 0.74 log10 CFU/mL compared to the initial inoculum at 72 h. The single drug of MRX-8 at 1.5 mg/L (MRX-8_3, corresponding to 0.75 mg/kg q8 continuous infusion) showed bacterial regrowth of 1 and 3 log10 CFU/mL increase than the initial inoculum at 24 and 72 h, respectively. It suggested that the single MRX-8 regimen had a rebound in bacterial colonies within 3 days. The combination of MRX-8 at 1.5 mg/L and meropenem at 8.77 mg/L (COM_2, corresponding to 0.75 mg/kg q8 continuous infusion of MRX-8 and 1 g q6h continuous infusion of meropenem) could kill the bacteria to the lower detection limit in the first 3 h and achieved bacteriostatic killing (decreased 0.03 log10 CFU/mL) compared to the initial inoculum at 72 h. The COM_2 could exhibit a 3.03 log10 CFU/mL reduction compared to MRX-8_3 at 72 h, which demonstrated a synergistic effect.

For AB21-3881, the MRX-8_1 and the COM_1 regimens could inhibit bacterial growth and maintained colony counts at the initial inoculum until 72 h. The COM_1 regimen demonstrated limited antibacterial effects (0.68 log10 CFU/mL decrease) compared to the MRX-8_1 regimen. The MRX-8_3 regimen showed manifest bactericidal effect by killing the bacteria to the lower detection limit at the first 3 h and maintained the bactericidal effect until 72 h by decreasing 3.9 log10 CFU/mL compared to the initial inoculum. The meropenem 2 g q12h regimen did not show any antibacterial effect. Since the MRX-8_3 regimen showed a bactericidal effect, there was no need to test the COM_2 regimen.

The AUBC was calculated for both strains to evaluate the bacterial killing effect, as shown in Fig. 3C and D. For AB21-3759, the AUBC only decreased with MRX-8 increasing dose within 24 h. The combinational regimens had a better bactericidal effect as multiple dosing was administered, as the AUBC decreased after 24 h significantly compared with the corresponding MRX-8 regimens. For AB21-3881, there was no significant difference between the administration regimen of MRX-8_1 and the COM_1 regimen. The MRX-8_3 regimen showed the lowest AUBC with bactericidal effect.

### Population analysis profile in HFIM

**Fig 4:**
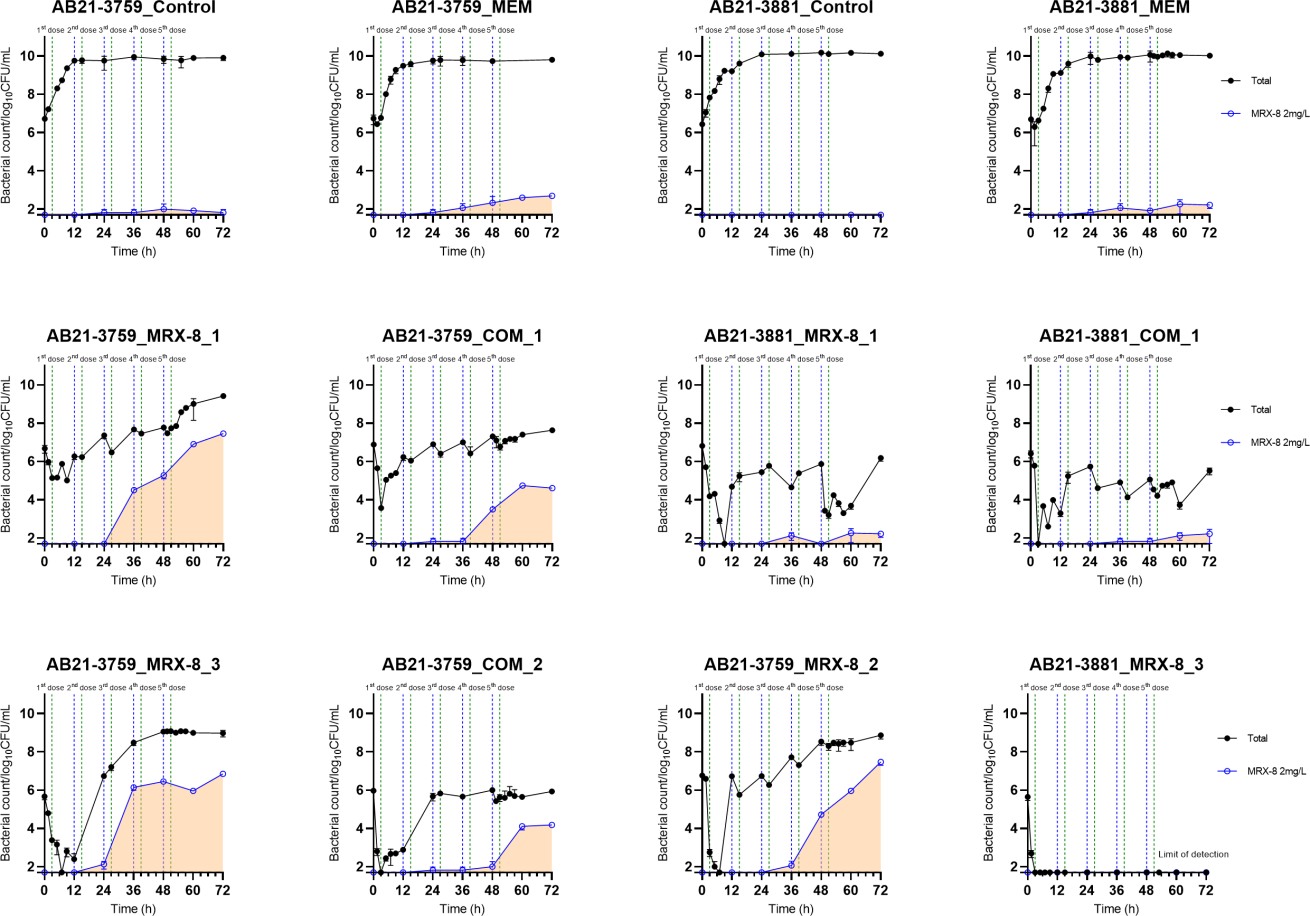
PAP analysis of AB21-3759 and AB21-3881: the black dot and line indicate the total bacterial colony counts; the blue square and line indicate the bacterial colony counts on the plate with 2 mg/L MRX-8. The blue dashed line indicates the start of dosing; the green dashed line indicates the completion of the dosing.

The results of the sub-population of adaptive resistant bacteria against MRX-8 in HFIM are shown in Fig. 4. Not surprisingly, the control and meropenem treatment alone did not induce the development of MRX-8 resistance for both strains. The administration of the MRX-8 regimen alone could induce adaptive resistance from 24 h for AB21-3759. The combination regimens could slow down the development of adaptive resistance by 12–24 h compared to the corresponding MRX-8 alone treatment for AB21-3759. For AB21-3881, the strain showed less adaptive resistant sub-population under both MRX-8 alone and combination treatment.

## DISCUSSION

Meropenem has demonstrated synergistic effects when combined with polymyxins against *A. baumannii in vitro* (11, 17, 18). Lenhard et al. reported that, even for bacteria with high meropenem MIC, increasing the meropenem dose and combining with polymyxin B resulted in synergistic bactericidal effect (19). The checkerboard assay results showed an additive or synergistic effect for the combination of meropenem and MRX-8 against seven different *A. baumannii* isolates. Subsequently, two isolates, which had exhibited synergistic and additive effects in the checkerboard assay, were selected for further static time-kill curve analysis and HFIM studies. These two isolates belong to the same sequence type (ST) and had *bla*_OXA-23_ as their primary carbapenem resistance mechanism.

The combination of MRX-8 and meropenem against *A. baumannii* demonstrated a synergistic effect in checkerboard assays. The results of static time-kill curve studies confirmed the synergistic and additive effects of the combination against the two strains. Moreover, it showed that if MRX-8 alone achieved sufficient bacterial killing, the synergistic effects were limited, and combinations were not necessary. The HFIM, which could evaluate bactericidal effect by simulating pharmacokinetic profiles of different regimens, was necessary to further validate the synergistic effect and optimize dosing regimens. HFIM results revealed that the combination regimens of COM_2 showed a synergistic effect at 72 h for AB21-3759 compared to MRX-8_2, despite it only achieving bacteriostasis compared to the initial inoculum. However, strain AB21-3881 did not show a significant advantage with the combination regimen. Since the two bacterial strains had the same MICs of meropenem and MRX-8 and enharboured similar bacterial resistance mechanisms, PAP analysis of the adaptive resistant sub-populations helped to explain this difference. The adaptive resistant sub-population profiles varied significantly among different regimens for the two strains. For AB21-3759, these sub-populations emerged within 24 h during MRX-8 monotherapy, and bacterial growth persisted despite multiple doses given every 12 h. Combinational therapy effectively inhibited the growth of this adaptive resistant sub-population, demonstrating a synergistic effect against the resistance development of AB21-3759. In contrast, AB21-3881 did not easily develop such sub-populations under any regimen, suggesting intrinsic differences in bacterial adaptation and stress response mechanisms (20, 21). Bacterial adaptation strategies, such as heteroresistance to polymyxins and biofilm formation, may explain the differential responses between the two strains (22, 23). Further studies are required to elucidate the mechanisms behind these differences, particularly for strains with identical drug susceptibility profiles. This study demonstrated that combination therapy can slow the development of adaptive resistance compared to single polymyxin treatment.

Clinical guidelines recommend meropenem at 2 g as a 3 h infusion every 8 h for bacteria with MIC ≤ 8 mg/L. In the present study, meropenem administered as a 2 g 3 h infusion every 12 h or as a 1 g q6h continuous infusion was tested alone and in combination. The daily dose of meropenem tested was not the highest dose yet. Increasing the meropenem dose in combination with MRX-8 may further enhance bactericidal effects for other clinical isolates, warranting additional *in vitro* and *in vivo* investigations.

### Conclusion

The results demonstrated that the combination of MRX-8 and meropenem exhibited both synergistic and additive effects in treating *A. baumannii* infections in checkerboard and static time-kill curves. HFIM studies showed that MRX-8 at 1 mg/kg q12h combined with meropenem at 2 g q12h, as well as MRX-8 at 0.75 mg/kg q8h continuous infusion combined with meropenem at 1 g q6h continuous infusion, achieved bacteriostatic killing at 72 h compared to the initial inoculum. The combination therapy could exert a synergistic effect by suppressing the emergence of adaptive resistance sub-populations compared to MRX-8 monotherapy, highlighting its potential to overcome resistance development.
